# Nano-Scale Spatial Assessment of Calcium Distribution in Coccolithophores Using Synchrotron-Based Nano-CT and STXM-NEXAFS

**DOI:** 10.3390/ijms151223604

**Published:** 2014-12-18

**Authors:** Shiyong Sun, Yanchen Yao, Xiang Zou, Shenglan Fan, Qing Zhou, Qunwei Dai, Faqin Dong, Mingxue Liu, Xiaoqin Nie, Daoyong Tan, Shuai Li

**Affiliations:** 1 Department of Geological and Mineral Engineering, Key Laboratory of Solid Waste Treatment and Resource Recycle & Fundamental Science on Nuclear Waste and Environmental Security Laboratory, Southwest University of Science and Technology, Mianyang 621010, China; E-Mails: shysun@swust.edu.cn (S.S.); yyc930124@163.com (Y.Y.); zxiang3354@163.com (X.Z.); shenglanfan@163.com (S.F.); kingchou1314@163.com (Q.Z.); qw_dai@163.com (Q.D.); dragonlmx@126.com (M.L.); xiaoqin_nie@163.com (X.N.); tdyduff@hotmail.com (D.T.); zmclishuai88@163.com (S.L.); 2 State Environmental Protection Key Laboratory of Microorganism Application and Risk Control (SMARC), Tsinghua University, Beijing 100084, China; 3 State Key Laboratory of Marine Geology, Tongji University, Shanghai 200092, China

**Keywords:** biomineralization, Nano-CT, STXM-NEXAFS, geobiology, coccolithophores

## Abstract

Calcified coccolithophores generate calcium carbonate scales around their cell surface. In light of predicted climate change and the global carbon cycle, the biomineralization ability of coccoliths has received growing interest. However, the underlying biomineralization mechanism is not yet well understood; the lack of non-invasive characterizing tools to obtain molecular level information involving biogenic processes and biomineral components remain significant challenges. In the present study, synchrotron-based Nano-computed Tomography (Nano-CT) and Scanning Transmission X-ray Microscopy-Near-edge X-ray Absorption Fine Structure Spectromicroscopy (STXM-NEXAFS) techniques were employed to identify Ca spatial distribution and investigate the compositional chemistry and distinctive features of the association between biomacromolecules and mineral components of calcite present in coccoliths. The Nano-CT results show that the coccolith scale vesicle is similar as a continuous single channel. The mature coccoliths were intracellularly distributed and immediately ejected and located at the exterior surface to form a coccoshpere. The NEXAFS spectromicroscopy results of the Ca L edge clearly demonstrate the existence of two levels of gradients spatially, indicating two distinctive forms of Ca in coccoliths: a crystalline-poor layer surrounded by a relatively crystalline-rich layer. The results show that Sr is absorbed by the coccoliths and that Sr/Ca substitution is rather homogeneous within the coccoliths. Our findings indicate that synchrotron-based STXM-NEXAFS and Nano-CT are excellent tools for the study of biominerals and provide information to clarify biomineralization mechanism.

## 1. Introduction

Calcified coccolithophores such as *Emiliania huxleyi* and *Pleurochrysis carterae*, are unicellular eukaryotic phytoplankton, enclosed by calcified scales called coccoliths [1]. Coccolithophores play a significant role in the marine carbon cycle by conversion processes of inorganic carbon (carbon dioxide) to organic compounds of living cells and biogenic inorganic minerals of calcite. The primary constituents of coccoliths are mineral calcite and acidic polysaccharides [2,3,4]. The formation of coccoliths is highly regulated by a dominant process known as biomineralization. Generally, biomineralization occurs in the Golgi-derived vesicles where protein templates nucleate the calcite crystals and coccolith associated polysaccharides (CPs) mediate the morphology and growth of these crystals [1,3]. As each scale is produced, it is secreted in a coccolith vesicle and tightly attached to the exterior cell surface and formed coccosphere [1,4,5]. Despite recognition of this biomineralization process, fundamental aspects of coccolith production have not been well demonstrated. A key approach to understand coccolith formation is integrating state-of-the-art microscopy techniques to acquire high-resolution spatial information at the nano-scale [6,7].

In the last decades, significant progress has been achieved in studying biogenic mineralization processes using synchrotron-based techniques such as Scanning Transmission X-ray Microscopy (STXM), Near-edge X-ray Absorption Fine Structure Spectromicroscopy (NEAFS) and Nanocomputed Tomography (Nano-CT) [8,9,10,11,12]. Compared to conventional microscopy techniques, synchrotron-based X-ray microscopy has advantages in highly penetrating depth and nanometer scale [9]. Therefore, the objective of the present study was to investigate the spatial distribution of inorganic mineral components within coccoliths to better understanding the biomineralization processes of coccolithophores by non-destructive state-of-the-art synchrotron-based X-ray microscopy techniques with high spatial information at nano-scale resolution.

## 2. Results and Discussion

**Figure 1:**
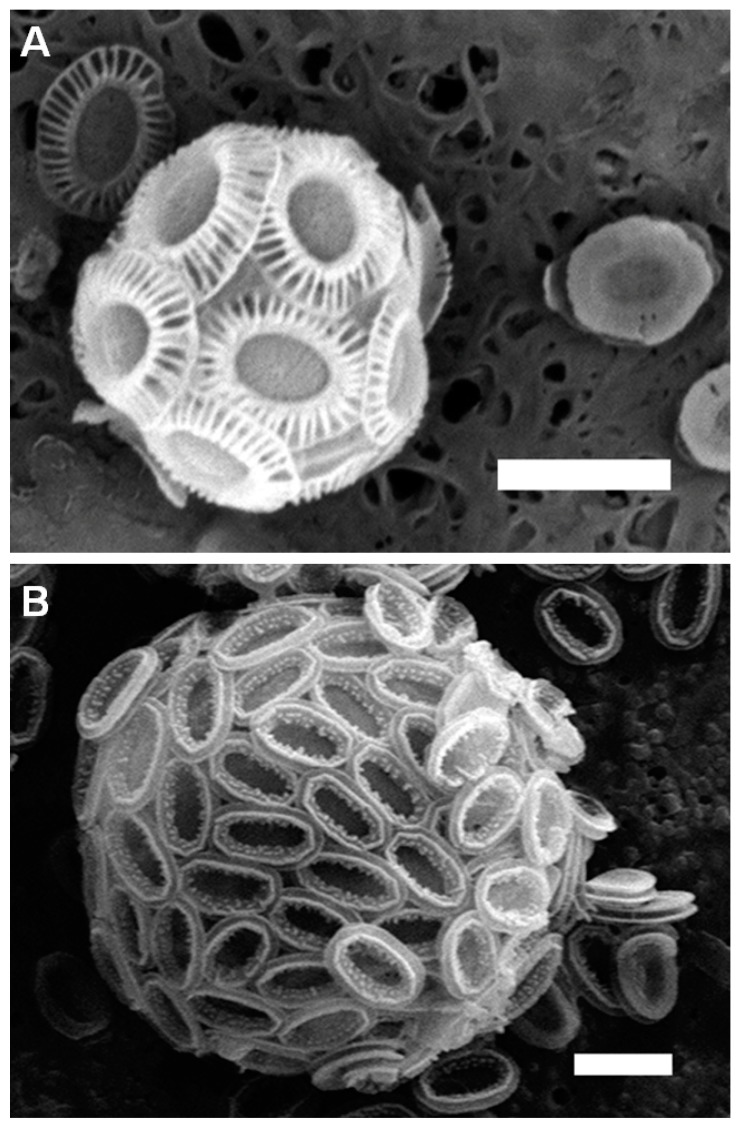
Scanning electron microscopyimages of typical morphology features of coccolithophores. (**A**) Image of a whole cell of *E. huxleyi*; (**B**) Image of a whole cell of *P. carterae*. The complete coccoliths in distal shield and proximal shield views are also presented in (**A**). Scale bars: 2 μm

The common morphological features of typical coccolithophores used in our studies were generally consistent with those published in the literature (Figure 1) [3,4,5]. As shown in Figure 1, the exterior cell surface was covered by coccolith scales that are embedded in a thin organic layer. One of the most distinctive aspects is that two species display very different morphological patterns.

### 2.1. Spatial Distribution of Coccoliths

**Figure 2:**
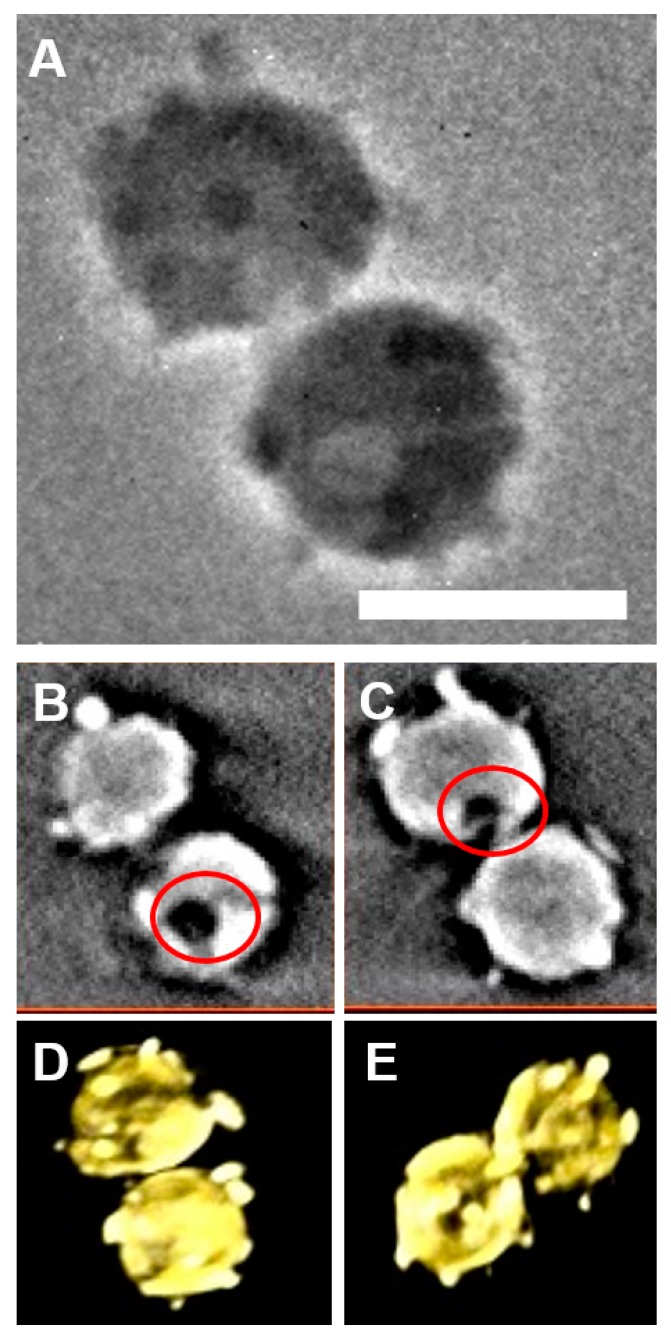
Different images of a single cell of *E. huxleyi*. (**A**) TXM 2D projection image; (**B,C**) representative two slices of reconstructed volume data showing different regions of cells; (**D,E**) representative two images of 3D rendering of reconstructed volume data with different rotation angles. Red color circles in (**B,C**) show the possible pore of the channel of the coccolith vesicle used for excreting coccolith scales. Scale bar in (**A**): 4 μm.

The different images of two *E. huxleyi* cells are shown in Figure 2. Figure 2A presents one original 2D projection image of *E. huxleyi*, in which the distinctive coccolith features are detectable. The representative two slices of reconstructed volume data show the presence of intracellular pores from different direction and regions of selected cells, indicating the pores are not caused by incident irradiation of the light source (Figure 2B,C). 3D rendering of reconstructed volume data with different rotation angles shows that coccoliths are distributed in exterior layers on the cell surface (Figure 2D,E).

A single large Golgi body and the reticular body, distinct from the Golgi body but thought to be derived from it, were always visible on the distal surface of the developing coccolith vesicles, suggesting this is a common structural feature that mineralizes and secretes coccoliths [1,4]. Time-lapse imaging confirmed that the coccolith secretion process is a rapid and retractile process that releases the coccoliths on the exterior surface of the cell [1]. It seems that the coccolith vesicle for excreting coccolith scales is roughly a single continuous channel, which was confirmed by intracellular tomographic sections showing variable pores in different direction (Figure 2B,C). Figure 2D,E show coccoliths plates are always observed on the exterior surface, probably as a result of mature coccoliths being pushed through the coccolith vesicle in a relatively rapid sequence to form the coccosphere.

### 2.2. Ca L Edge of Coccoliths

**Figure 3:**
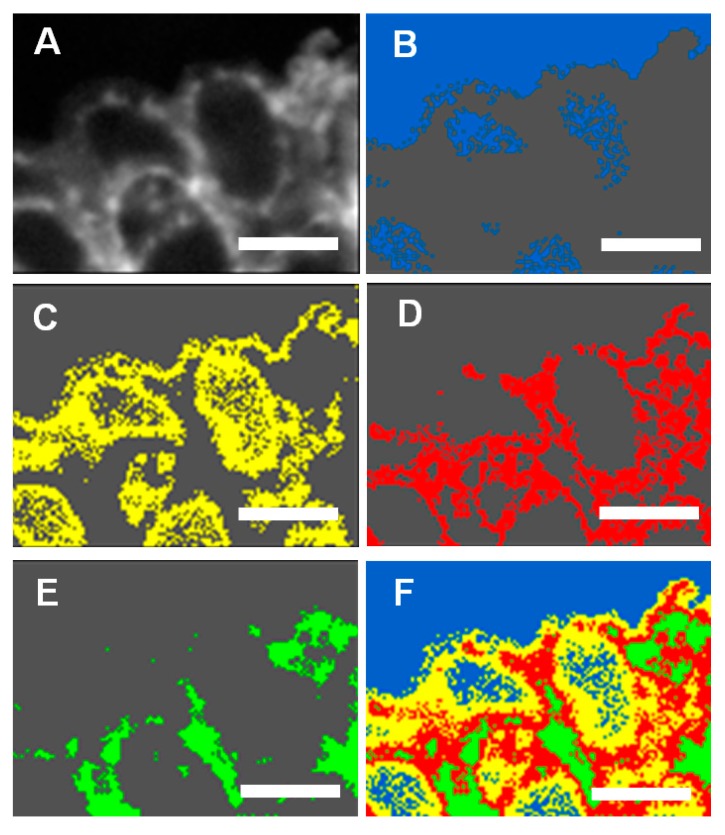
High-resolution (50 nm) spatial distribution of total Ca in *E. huxleyi* determined by STXM-NEXAFS spectromicroscopy. (**A**) Contrast tomography image; (**B**) Cluster 1 of background with blue color; (**C**) Cluster 2 of Ca form with yellow color; (**D**) Cluster 3 of Ca form with red color; (**E**) Cluster 4 of Ca form with green color; (**F**) Merged images of (**B–E**), Scale bars: 1 μm.

**Figure 4:**
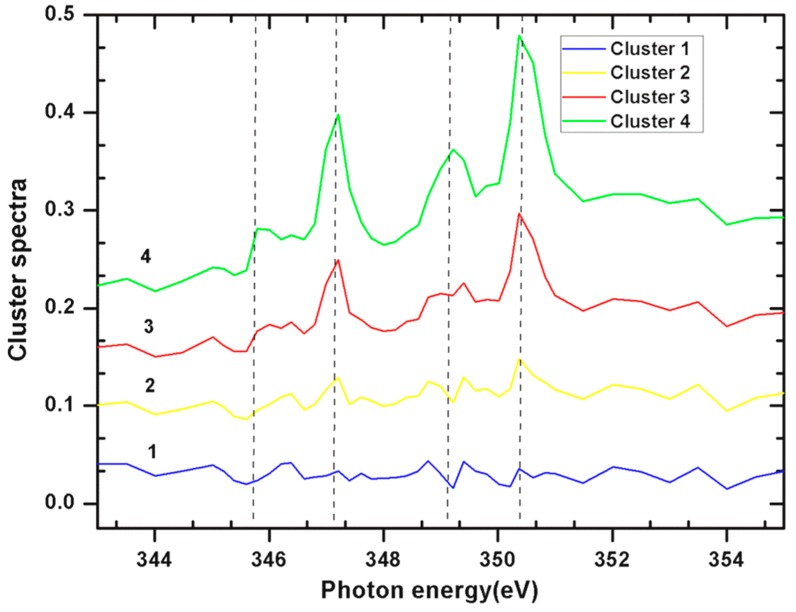
NEXAFS spectra of Ca L_2,3_ edge of total Ca in *E. huxleyi* by cluster measurements of images as showed in Figure 3.

**Figure 5:**
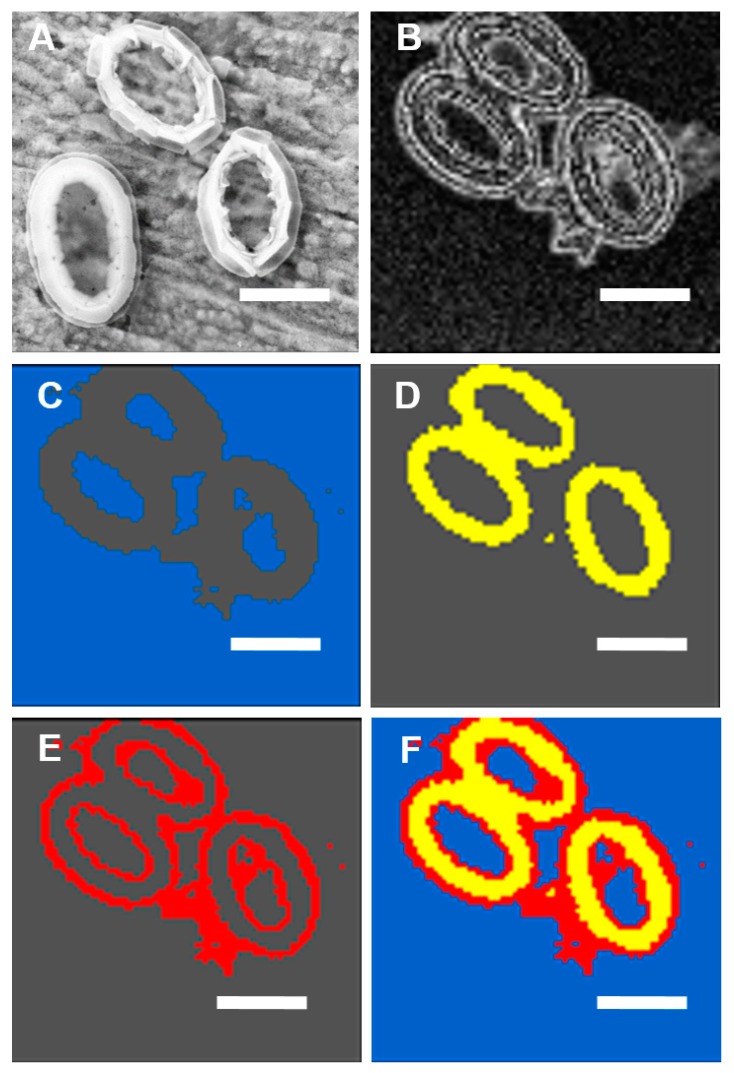
High-resolution (50 nm) spatial distribution of total Ca in *P. carterae* determined by STXM-NEXAFS spectromicroscopy. (**A**) Coccoliths image using FE-SEM; (**B**) Contrast tomography image; (**C**) Cluster 1 of background with blue color; (**D**) Cluster 2 of Ca form with yellow color; (**E**) Cluster 3 of Ca form with red color; (**F**) Merged images of (**C**–**E**). Scale bars: 2 μm.

**Figure 6:**
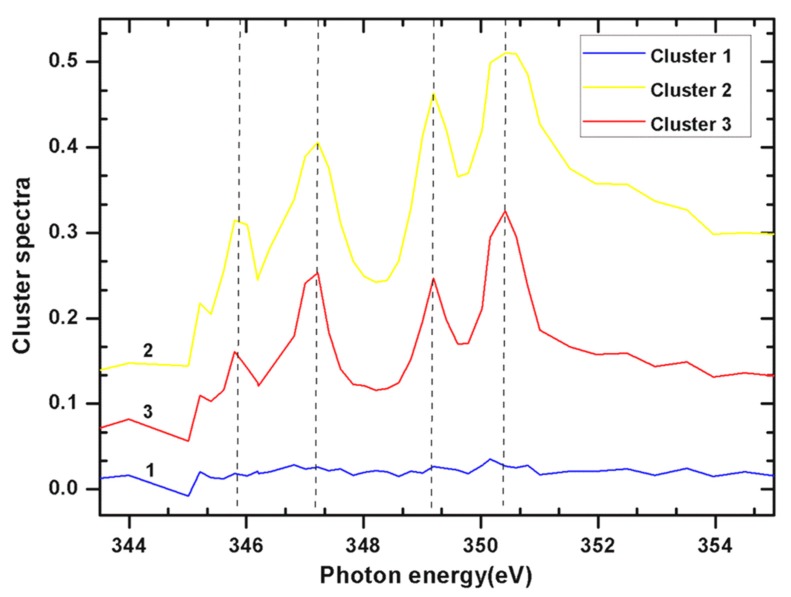
NEXAFS spectra of total Ca in *P. carterae* by cluster measurements of images as showed in Figure 5.

The spatially resolved Ca L edge STXM-NEXAFS spectromicroscopy images of *E. huxleyi* (Figure 3 and Figure 4) and *P. carterae* (Figure 5 and Figure 6) show contrasting images of distribution of total Ca and Ca L-edge NEXAFS spectra within intact coccoliths with fine resolution (50 nm). Further analyses show that there are spatially distinct regions within coccoliths that have highly variable Ca gradients (Figure 3 and Figure 5). *In situ* Assessment of spatial heterogeneity of coccoliths shows existing highly variable gradients of Ca in three forms from *E. huxleyi,* and two forms from *P. carterae* (Table 1); variations in thickness are often classified as distinct regions by cluster method [13]. As shown in Figure 3, cluster 4 appears to be the overlapped layers of cluster 2 and cluster 3. Therefore, the associations Ca forms in coccoliths of *E. huxleyi* and *P. carterae* are typically categorized into two levels. It has been shown that the central area of complete coccolith is a product of less regular growth [4]. It seems that these two levels of distinctive clusters of coccoliths present a crystalline-poor layer surrounded by a relatively well crystalline-rich layer.

As shown in Figure 4 of *E. huxleyi* and Figure 6 of *P. carterae*, all spectra of coccoliths consist of the two main spin-orbit L_2,3_ related peaks (L_3_2P_3/2_ at 347.19 eV and L_2_2P_1/2_ at 350.37 eV) along with smaller peaks of L_3_ (345.82 eV) and L_2_ (349.19 eV), respectively [14]. The position of these multi-peak patterns is known to be site-symmetry of Ca as well as the crystal field splitting [15]. It has been reported that the smaller peaks of L_3_ and L_2_ are sensitive to the symmetry of the atoms surrounding the Ca^2+^ ion in the first coordination sphere [14]. Although no definitive structural information about the Ca forms could be quantitatively extracted at this time, it seems that the presence of inorganic and intermediate forms of biomacromolecule-Ca compounds were included in these coccoliths.

**Table 1:** Total Ca of pixels in cluster percentages in coccoliths by cluster measurements of images as showed in Figure 3 and Figure 5.

Cluster Index	Pixels in Cluster %	Pixels in Cluster %
	*Emiliania* *huxleyi*	*Pleurochrysis carterae*
Cluster 1	33.949	70.5536
Cluster 2	30.1769	15.0029
Cluster 3	24.3236	14.4435
Cluster 4	11.5505	

### 2.3. Sr/Ca Substitution

**Figure 7:**
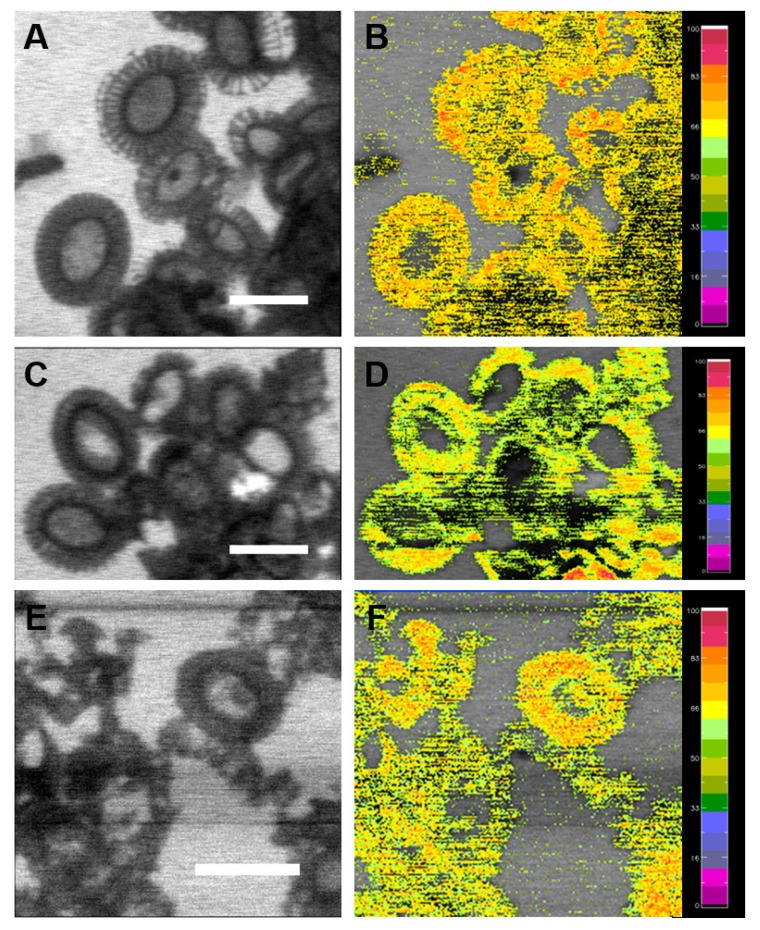
Dual energy (347.7 and 345 eV) contrast images of total Ca in coccoliths of *E. huxleyi* in the presence of different Sr concentrations using STXM. (**A,B**) and (**B**) 0 ppm Sr in original culture medium; (**C,D**) 50 ppm Sr in original culture medium; (**E,F**) 100 ppm Sr in original culture medium; (**A,C,E**) Ca mapping at 347.7 eV; (**B,D,F**) Color overlay from the difference of 347.7 and 345 eV images showing the specific distribution of Ca with gradients. Scale bars: 1 μm.

STXM is an excellent tool for mapping specific elemental composition with high (50 nm) spatial resolution. Chemical specific mapping was derived from dual energy images taken at specific absorption energy and away from absorption energy [7,11]. Dual energy (347.7 and 345 eV) contrast images of total Ca in coccoliths of *E. huxleyi* are illustrated in Figure 7. Figure 7A,C,E can be interpreted as a non-specific distribution of the Ca content. Figure 7B,D,F show more specific Ca images and represent an elemental mapping of the total Ca constructed from the different images at absorption energy of Ca L edge of 347.7 eV and away from absorption energy of 345 eV. Small amounts of Ca were heterogeneously distributed in the coccoliths with the presence of 50 ppm Sr, which can be interpreted as overlapped layers of coccoliths (Figure 7D). SEM-EDS quantifications of atomic ratios of Ca, Mg and Sr elements from coccoliths of *E. huxleyi* show that the substitution ratio of Sr/Ca is increased with exposure to higher concentration of Sr in initial culture medium (Table 2). Although, the substitution ratio of Sr to Ca increased, it performed in a non-specific manner that homogenous distribution in a complete coccolith.

**Table 2:** SEM-EDS quantifications of atomic ratios of Ca, Mg and Sr elements in coccoliths of *E. huxleyi* in the presence of different Sr concentrations in original culture mediums.

Original Sr	Ca	Mg	Sr	Sr/Ca	Mg/Ca
ppm	%	%	%		
0	5.3	0.43	0.02	0.004	0.081
50	6.21	1.72	0.11	0.018	0.277
100	6.78	3.28	0.23	0.034	0.484

## 3. Experimental Section

### 3.1. Culture Conditions

The Coccolithophore of *E. huxleyi* (CS-369) was originated from CSIRO Victoria, Australia). The Coccolithophore of *P. carterae* was originated from CCMP (East Boothbay, ME, USA). Aquil medium recipie was used for preparing culture medium by analytical grade chemical salts [16]. The cell cultures were maintained in a light incubator at a constant temperature of 25 °C under light exposure of 20,000 lux in 12 h:12 h (light:dark) cycles. Strontium nitrate was added in the initial culture medium with final concentrations of 0, 50 and 100 ppm, respectively. The cell concentrations were counted under a light microscope using a hemocytometer. The cultured cells in stationary phase were collected by centrifugation for further experiments.

### 3.2. Nano-CT

The collected samples were observed by FE-SEM (Ultra55, Zeiss, Oberkochen, Germany) in the Analysis Center of Southwest University of Science and Technology before Nano-CT and STXM-NEXAFS investigations. The Nano-CT experiments were performed on the U7A beamline of National Synchrotron Radiation Laboratory (NSRL) in Hefei, China [17]. A 6T superconducting wiggler is used as the X-ray source. A Si (111) double-crystal monochromator tunes the photo energy of 7–12 keV to provide a monochromatic X-ray flux. For 3D reconstruction, the 151 sequential tomographic images were collected at 1° intervals from −75° to +75° at 8 keV with a field view of 15 × 15 μm in a spatial resolution of 60 nm [9,17]. These projections were subsequently aligned, and a standard filtered-back-projection algorithm was used to reconstruct the aligned data [17].

### 3.3. STXM-NEXAFS

The Ca L_2,3_ edge STXM–NEXAFS spectroscopy measurements were carried out with the soft X-ray spectromicroscopy beamline (BL08UA) of the Shanghai Synchrotron Radiation Facility [18,19]. The samples were resuspended in ethanol and dropped on a silicon nitride window (Shanghai NTI Co., Ltd., Shanghai, China) before being mounted onto the sample holder of the beamline. A sequence of stack images around Ca L_2,3_ edge were collected at energies of 342–360 eV with 0.1 eV energy steps in dwell time of 1 s. The cluster analysis method was used for obtaining spatial distribution of Ca speciation and simultaneously also the corresponding NEXAFS spectra [20]. NEXAFS spectra were extracted from groups of pixels with similar absorption features using the IDL package aXis2000. These spectra are called cluster spectra corresponding to the specific regions of interested specimens. The transmission intensities from clean areas of the silicon nitride membrane were used as incident intensities for normalization of the transmission signals obtained from the areas of interest.

## 4. Conclusions

Our study demonstrated that synchrotron-based X-ray spectromicroscopy and tomography investigations can provide in-depth spatial information of coccolithophores of various compositions and structures with resolution at the nanoscale level. The *in situ* spatial distributions of Ca in coccoliths clearly indicate the intimate association between the inorganic mineral of calcite and biomacromolecules. Particularly of interest, a periodic distribution with two gradient levels of Ca is present in coccoliths. The synchrotron-based microscopy techniques will be applied in studying biomineralization mechanism in biogenic minerals in living systems.
